# Endovascular Treatment of Right Heart Masses Utilizing the AngioVac System: A 6-Year Single-Center Observational Study

**DOI:** 10.1155/2021/9923440

**Published:** 2021-10-31

**Authors:** Aashish Katapadi, Lauren Richards, William Fischer, Suhail Q. Allaqaband, Tanvir Bajwa, M. Fuad Jan

**Affiliations:** Aurora Cardiovascular and Thoracic Services, Aurora Sinai/Aurora St. Luke's Medical Centers, Advocate Aurora Health, 2801 W. Kinnickinnic River Parkway, Ste. 880, Milwaukee, Wisconsin 53215, USA

## Abstract

**Objective:**

To describe our institution's experience with the AngioVac system.

**Background:**

Intracardiac and intravascular masses previously required surgical excision, but now, there are a number of minimally invasive options. With the advent of vacuum aspiration, more specifically the AngioVac System (AngioDynamics, NY, USA), there exists a system with both low mortality and minor complications. However, the number of retrospective studies remains limited. Outcome data for high-risk patients are also limited.

**Methods:**

Data were collected and analyzed in patients who underwent AngioVac therapy at our tertiary care center from January 2014 to December 2020.

**Results:**

Our results demonstrated a 93.3% intraoperative success rate and a 100% intraoperative survival rate. However, a number of complications, including but not limited to hematomas, anemia, and hypotension, occurred, as described below.

**Conclusions:**

Our experiences demonstrated good outcomes and continue to support the usefulness of the AngioVac System. The data also support the use of AngioVac as a treatment option for the debulking or removal of right heart masses in critically ill patients.

## 1. Introduction

Intracardiac masses, specifically right-sided cardiac thrombi, vegetations, and tumors, can be rare and life-threatening conditions and, moreover, are often difficult to manage. Mortality rates are often high in untreated patients [1, 2]. Treatment options for these patients traditionally include catheter-directed or systemic thrombolysis, embolectomy, and medical management alone. Catheter-based suction embolectomy has been successfully used for minimally invasive treatment of intravascular material. One such option is the AngioVac System (AngioVac Cannula and Circuit, AngioDynamics, NY, USA), a vacuum-assisted suction embolectomy device designed to remove fresh thrombi and vegetations in the right atrium and ventricle, superior and inferior vena cavae, and iliofemoral veins [3, 4]. The system has existed for a number of years, and its utility has been evaluated in a number of case studies [5–7]. However, data from recent clinical studies have remained limited or nonexistent. Additionally, the AngioVac System has been considered for off-label use for a variety of conditions including as an alternative to surgical thrombectomy in high-risk patients for the removal or debulking of infectious vegetations and benign or malignant tumors in the right atrium, ventricle, or tricuspid valve. There are limited data regarding outcomes for these patients. Consequently, the purpose of this report is to describe our institution's experiences and outcomes with the AngioVac System over the past 6 years.

## 2. Materials and Methods

The local institutional review board approved this study and waived the requirement for informed consent.

### 2.1. Study Population

All cases of AngioVac-assisted, catheter-based extraction techniques performed in the cardiac catheterization laboratory at our large tertiary care center from 2014 to 2020 were reviewed. The decision to proceed with AngioVac resulted from collaboration between multiple specialties (interventional cardiology, infectious diseases, and intensive care physicians) and the patient; stability of the patient was also a consideration.

### 2.2. AngioVac System

**Figure 1:**
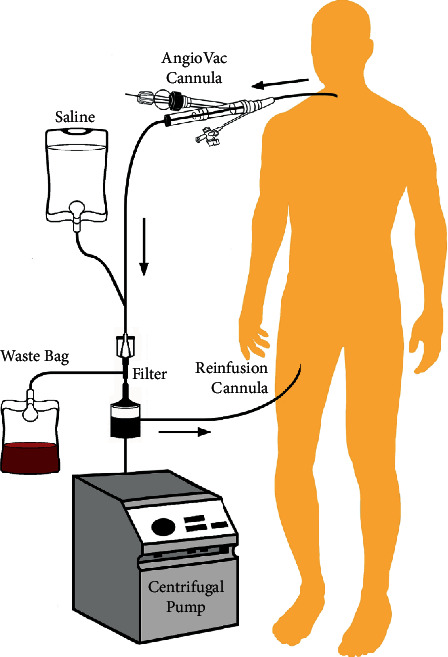
An overview of the AngioVac reperfusion system. The tip is inserted either via percutaneous or surgical cutdown at the internal jugular or femoral vein and connected to a suction-generating bypass circuit. Blood is filtered and reinserted at the reinfusion cannula.

The AngioVac System is a disposable intravenous system that is used with extracorporeal circulatory support. It can be used to remove fresh, soft thrombi, emboli, or vegetations in the right atrium and ventricle, superior and inferior vena cavae, and iliofemoral veins. The system consists of a 22-F AngioVac Cannula with a self-expanding tip that is used in conjunction with a reinfusion cannula as part of a bypass circuit (Figure 1). A centrifugal pump enables suction at the tip, which, when deployed through either the internal jugular or femoral veins, enables removal of undesirable intravascular material.

The system requires two venous access sites—1 for aspiration and 1 for reperfusion—usually utilizing a combination of femoral or jugular veins. The extracorporeal bypass circuit consists of an outflow line, a centrifugal pump, a filter, and an inflow line. After venous access is obtained, the centrifugal pump is activated, creating a one-way flow that provides suction at the cannula tip. The system has a balloon-activated tip to augment venous flow and facilitate removal of the thrombogenic material into the filter. The circuit reinfuses filtered blood back into the body through the reperfusion cannula to minimize blood loss (Figure 1).

### 2.3. Data Collection

Retrospective data from each patient, including demographics, medical history, treatment, procedural indication, procedural variables, imaging data (transthoracic/transesophageal echocardiogram, computed tomography, and magnetic resonance imaging), and both postoperative and long-term outcomes, were recorded. Importantly, intraoperative success was defined as complete removal of the mass or removal of >50% of the clot as seen on intraprocedural transesophageal echocardiography and per procedure note. Postprocedural echocardiography was reviewed when available.

### 2.4. Statistical Analysis

Results were reported for both quantitative and categorical variables. Results for quantitative variables were reported as median with interquartile range, and those for categorical variables were reported as frequencies and percentages.

## 3. Results

### 3.1. Preoperative Characteristics

Demographic and preoperative variables are listed in Tables 1 and 2, respectively. The procedure was performed on a total of 17 patients, 10 females and 7 males, with an average age of 47.1 years. Indications for AngioVac were thrombus (35.3%, *n* = 6/17), septic thrombus (5.9%, *n* = 1/17), and endocarditis (58.8%, *n* = 10/17). All patients were critically ill with high surgical risk. The average size of thrombus or vegetation was 3.48 cm, all located on the tricuspid valve (47.1%, *n* = 8/17) and right atrium with or without vena cava involvement (41.2%, *n* = 7/17). A number of masses were mobile (64.7%, *n* = 11/17). Patients with endocarditis grew methicillin-susceptible *Staphylococcus aureu*s (MSSA; 29.4%, *n* = 5/17), methicillin-resistant *Staphylococcus aureus* (MRSA; 17.6%, *n* = 3/17), *Staphylococcus epidermidis* (5.9%, *n* = 1/17), and *Enterococcus faecalis* (5.9%, *n* = 1/17). Pathologic examination of the extracted mass was not performed to confirm diagnosis (Tables 1 and 2).

### 3.2. Postoperative Characteristics

Postoperative variables are listed in Table 3. The procedure was successful in almost all of the patients (94.1%, *n* = 16/17), failing only once, in a patient with infective endocarditis. Almost all patients (82.4%, *n* = 14/17) survived to discharge and to 30 days after hospitalization (76.5%, *n* = 13/17). The 3 patients who did not survive to discharge expired due to septic shock, severe metabolic derangements, and ruptured peptic ulcer leading to respiratory failure. Of the 14 discharged, 4 had expired at 1 year, and 6 had not met the time requirements; the 1-year survival decreased greatly (23.5%, *n* = 4/17). The leading cause of in-hospital death was shock, both septic (11.8%, *n* = 2/17) and cardiogenic (5.9%, *n* = 1/17); malignancy, more specifically advanced stage glioblastoma and colon cancer, was the cause of death in 2/17 who did make it to 1 year (11.8%). Hematoma developed in 3/17 (17.6%) after AngioVac use. After the procedure, a number of patients developed hypotension (35.3%, *n* = 6/17), with some requiring vasopressor support (11.8%, *n* = 2/17) and some requiring transfusion (29.4%, *n* = 5/17). Other complications included worsening tricuspid regurgitation (5.9%, *n* = 1/17), pulmonary embolism (5.9%, *n* = 1/17), and formation of mycotic aneurysm (5.9%, *n* = 1/17). The average postprocedural hospital length of stay was 8.07 days, and many returned home following their procedure (64.7%, *n* = 11/17).

## 4. Discussion

Strategies for intravascular or intracardiac masses traditionally revolve around anticoagulation, thrombolysis, and surgical interventions. In patients for whom thrombolysis is contraindicated, surgical therapy such as embolectomy is an option. The introduction of catheter-based rheolytic and aspiration technologies such as AngioVac offers an alternative, minimally invasive approach to thrombectomy with low morbidity. AngioVac, in particular, has the advantage of whole, intact thrombus aspiration. This approach also decreases the number of complications in an already high-risk patient population. Although AngioVac is US Food and Drug Administration-approved only for thrombi and emboli, this benefit makes it appealing for use with additional indications.

While the earliest literature referencing the AngioVac System describes the percutaneous extraction of a 1.7 cm right atrial mass in the setting of endocarditis, the device has been utilized on vegetations on implantable cardioverter-defibrillator leads and permanent pacemakers with a survival benefit [4, 8]. The variety of indications has continued to grow as cases have been added to the literature [9–11]. Patients treated for infective endocarditis with large vegetations (>10 mm) with clinical evidence of embolic phenomena are often critically ill and carry a high perioperative risk for open cardiac surgery [12]. Although AngioVac is not yet approved for the debulking of infectious vegetations, it has been used for patients in inoperable situations in which mortality is almost certain without further treatment.

Our own data are characterized by use of AngioVac in a similar population and in the cardiac catheterization laboratory. Procedural success and procedural survival in this population were 94.1% and 100%, respectively. Furthermore, survival to discharge and 30-day survival were 82.4% and 76.5%, respectively. Immediate success for these patients was high and provided an opportunity to prolong life in critically ill patients without increasing mortality. Out of all the patients, 1-year survival greatly decreased to 23.5%. However, it is important to note that 6 patients had not yet made it to 1 year and another 3 did not survive hospitalization. Of the 4 who passed away, 1 was due to progressive failure to thrive postprocedure, 1 was due to severe septic shock with bacteremia, and 2 were due to advanced cancers, as discussed previously. One procedure utilizing AngioVac was unsuccessful and later required surgery for an infected thrombus on an intracardiac lead. Only 2 patients did not survive to discharge. Of these 2, 1 passed away due to cardiogenic shock in the setting of severe tricuspid regurgitation and the other due to severe septic shock. Though both patients had moderate-sized masses and met intraoperative success, they remained critically ill following the procedures. The patients who did survive past 1 year appeared to do well with no sequelae of endocarditis. Unfortunately, many patients with infective endocarditis did relapse or continue using intravenous drugs. This highlights both the severity of substance abuse, especially intravenous drug use, and difficulties of treating this patient population.

Complications most noted were hematoma at the site of catheter insertion, acute anemia requiring transfusion, and persistent hypotension. Other notable complications, though only occurring in 1 patient each, were worsening tricuspid regurgitation, supraventricular tachycardia and sinus tachycardia, and pulmonary embolism and mycotic aneurysms.

**Figure 2:**
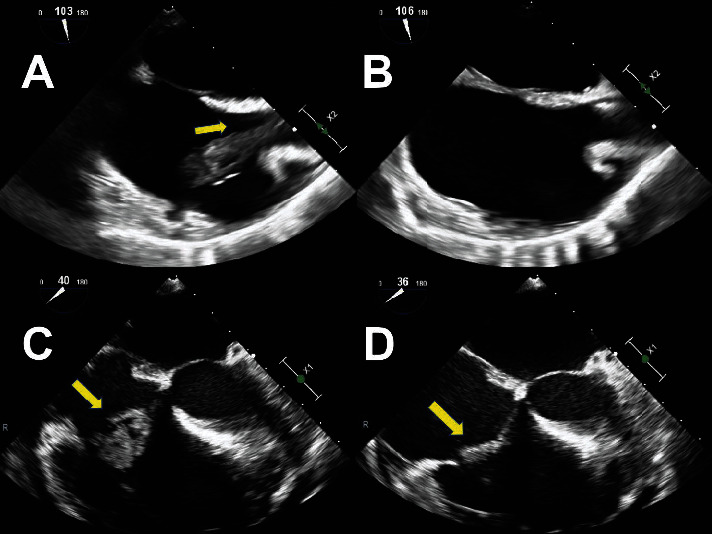
Pre- and postprocedural transesophageal echocardiographic imaging of the removal of large thrombi via AngioVac. (a, c) Large vegetation across the mitral valve. (b, d) Near removal of multisegmented vegetation. There is good intraoperative success.

There continues to exist a small number of retrospective studies involving the AngioVac System. More recently, a large registry with 234 concomitant procedures demonstrated the safety of the AngioVac System, with only 3 procedure-related deaths. The indication for the procedure was a right heart mass in 52.6% of cases; intraprocedural success, defined as >70% mass removal, was achieved in nearly 60% of patients with right heart masses [13]. Our own results coincide with previously published data, as seen in Table 4, demonstrating good intraoperative success [5, 7, 14–16]. Likewise, these results also support the use of AngioVac as a treatment option for critically ill patients with right-sided heart masses, as seen in Figure 2. Short-term success and survival rates are high. Information about long-term outcomes in those who undergo the AngioVac procedure does not currently exist. Patients with infective endocarditis have only 50% survival at 10 years, with the highest survival rate in people who undergo early surgery [17, 18]. The AngioVac procedure provides life-prolonging measures otherwise unavailable for patients who may otherwise not live to discharge. This supports the idea that vacuum-assisted thrombectomy, specifically AngioVac at this time, should be used in the algorithm for right-sided cardiac masses.

Despite promising results at 30 days, the data are limited by study size at a single center, and a larger patient population is necessary. Additionally, there are no prospective studies comparing surgery or medical management to AngioVac. Subsequently, more studies are required to determine if AngioVac has a mortality benefit over surgical or medical management.

## 5. Conclusion

The utility of the AngioVac System has been demonstrated in multiple case studies and in a few retrospective studies. We have presented the experience with the system at our institution, and though patients were critically ill with large vegetative masses, our data demonstrate good intraoperative survival and success. Our data appear to further support the use of AngioVac in the cardiac catheterization laboratory as a treatment option for right heart masses in critically ill patients with high surgical risk. Larger studies are required to determine safety in large vegetations of use with right-sided endocarditis.

## Figures and Tables

**Table 1:** Demographics.

Characteristics	*n* (%) or median (IQR)
*Sex*	
Male	7 (41.2)
Female	10 (58.8)
Average age (years)	47.1 (33–64)
BMI	28.1 (21.1–32.9)
*History*	
End-stage renal disease	1 (5.9)
Coronary artery disease	4 (23.5)
Valvular disease	2 (11.8)
Prior thrombotic disease	1 (5.9)
CHF	3 (17.6)
Diabetes	2 (11.8)
Hypertension	6 (35.3)
Malignancy	4 (23.5)
Iatrogenic immunosuppression	2 (11.8)
Prior cardiac devices	2 (11.8)

BMI: body mass index; CHF: congestive heart failure; IQR: interquartile range.

**Table 2:** Preoperative variables.

Indication	*n* (%) or median (IQR)
Thrombus	6 (35.3)
Septic thrombus	1 (5.9)
Endocarditis	10 (58.8)
Bacterial cultures	
Methicillin-sensitive *Staph*. *aureus*	5 (29.4)
Methicillin-resistance *Staph*. *aureus*	3 (17.6)
* Staph*. *epidermidis*	1 (5.9)
*E*. *faecalis*	1 (5.9)
Polymicrobial*∗*	2
Location*∗∗*	
SVC	1 (5.9)
IVC/RA	2 (11.8)
RA	5 (29.4)
TV	8 (47.1)
Average size	3.48 (2.25–4.7)
Mobility	
Mobile	11 (64.7)
Immobile	2 (11.8)
LVEF	51.9 (42–60)
Hemoglobin	9.1 (7.8–10.7)
Creatinine	1.12 (0.67–0.98)

^∗^Cultures include MRSA/*Strep. gordonii* and *Enterobacter*/*Klebsiella*/*Citrobacter*/*Bacteroides*. ^∗∗^Multiple locations documented. IQR: interquartile range; IVC: inferior vena cava; LVEF: left ventricular ejection fraction; MRSA: methicillin-resistant *Staphylococcus aureus*; RA: right atrium; *Staph*: *Staphylococcus*; *Strep*: *Streptococci*; SVC: superior vena cava; TV: tricuspid valve.

**Table 3:** Postoperative variables.

Variable	*n* (%) or median (IQR)
Operative success	16 (94.1)
Survival	17 (100.0)
Survival to discharge	14 (82.4)
30-day survival	13 (76.5)
One-year survival	4 (23.5)
Average LOS	8.07 (3.0–10.0)
Need for tPA	0 (0.0)
Need for transfusion	5 (29.4)
Need for hemodialysis	0 (0.0)
LVEF*∗*	57.5 (54.25–62.75)
Side effects*∗∗*	
Hematomas	3 (17.6)
Shock	1 (5.9)
Hypotension	6 (35.3)
Vasopressor use	2 (11.8)
Hemoglobin	8.7 (7.4–9.5)
Creatinine	1.09 (0.63–1.15)

^∗^Nine patients missing postoperative LVEF. ^∗∗^Other side effects were worsening tachycardia, PE, mycotic aneurysm, intraoperative SVT, need for intubation (2), and need for the IVC filter. IQR: interquartile range; IVC: inferior vena cava; LOS: length of stay; LVEF: left ventricular ejection fraction; PE: pulmonary embolism; SVT: supraventricular tachycardia; tPA: tissue plasminogen activator.

**Table 4:** Prior retrospective studies.

	Donaldson et al. [5]	Salsamendi et al. [14]	Al Badri et al. [7]	Rajput et al. [15]	Fallon et al. [16]	Moriarty et al. [13]
Year	2015	2015	2016	2020	2020	2021
Patients (*n*)	15	7	7	16	58	234
Mean age (years)	50	49.6	51.5	48	48	50.8
Female sex (*n*)	3	4	5	12	27	111
Indication	Caval thrombus (*n* = 11)	Caval thrombus (*n* = 2)	IVC-associated mass (*n* = 3)	Right-sided intracardiac mass (*n* = 11)	Sterile thrombus (*n* = 30)	Right-sided intracardiac mass (*n* = 123)
	Pulmonary embolism (*n* = 5)	Fontan conduit and Glenn shunt mass (*n* = 1)	Pulmonary embolism (*n* = 2)	Catheter-associated thrombus (*n* = 7)	Cardiac device-associated vegetation (*n* = 8)	Catheter-associated thrombus (*n* = 25)
	Catheter-associated thrombus (*n* = 2)	Intracardiac mass (*n* = 4)	Catheter-associated thrombus (*n* = 2)	Caval thrombus (*n* = 9)	Chronic vascular access-associated vegetation (*n* = 16)	Caval thrombus (*n* = 91)
				Concurrent pulmonary embolism (*n* = 7)	IVDU-related vegetation (*n* = 4)	Pulmonary embolism (*n* = 7)
						Left-sided intracardiac mass (*n* = 1)
Location	RA (*n* = 11)	Vena cava (*n* = 2)	RA into RV (*n* = 4)	RA (*n* = 11)	SVC (*n* = 17)	SVC (*n* = 13)
	RV (*n* = 3)	PA (*n* = 2)	IVC (*n* = 3)	Vena cava (*n* = 9)	RA (*n* = 31)	RA (*n* = 98)
		RA (*n* = 1)			TV/RV (*n* = 11)	TV (*n* = 14)
		IVC (*n* = 1)			PA (*n* = 1)	RV (*n* = 4)
		SVC (*n* = 1)			IVC (*n* = 24)	IVC (*n* = 1)
					Infrailiac (*n* = 8)	
Average size of the mass	NA	NA	NA	4.1 cm	3.2 cm	NA
Procedural success	11	5	6	13	14	182
Postprocedural complications	Bleeding (*n* = 11)	Hematoma (*n* = 3)	Cardiogenic shock (*n* = 1)	Transfusion without overt bleeding (*n* = 10)	Persistent or recurrent bacteremia (*n* = 11)	Bleeding (*n* = 9)
	Shock (*n* = 5)			Shock (*n* = 1)	Pulmonary embolism (*n* = 8)	Transfusion (*n* = 59)
	Hemodialysis (*n* = 3)			Hemodialysis (*n* = 2)	Persistent tricuspid regurgitation (*n* = 19)	Pulmonary embolism (*n* = 1)
	Liver failure (*n* = 2)					Persistent bacteremia (*n* = 1)
						Stroke (*n* = 1)
						Hematoma (*n* = 4)
						Arrhythmia (*n* = 3)
Average LOS	23	NA	20	13.82	16.84	NA
Intraprocedural survival	13	7	7	14	57	231
Survival to discharge	13	7	7	14	NA	NA

IVC: inferior vena cava; IVDU: intravenous drug use; LOS: length of stay; PA: pulmonary artery; RA: right atrium; RV: right ventricle; SVC: superior vena cava; TV: tricuspid valve.

## Data Availability

The data used to support the findings of this study are available from the corresponding author upon reasonable request.
